# Generation and Characterization of Alloantigen-Specific Regulatory T Cells For Clinical Transplant Tolerance

**DOI:** 10.1038/s41598-018-19621-6

**Published:** 2018-01-18

**Authors:** James M. Mathew, Jessica H. Voss, Scott T. McEwen, Iwona Konieczna, Arjun Chakraborty, Xuemei Huang, Jie He, Lorenzo Gallon, Richard S. Kornbluth, Joseph R. Leventhal

**Affiliations:** 1 Department of Surgery - Comprehensive Transplant Center, Northwestern University Feinberg School of Medicine, Chicago, IL USA; 2 Department of Microbiology-Immunology, Northwestern University Feinberg School of Medicine, Chicago, IL USA; 3 Department of Medicine- Nephrology, Northwestern University Feinberg School of Medicine, Chicago, IL USA; 4 Ann & Robert H. Lurie Children’s Hospital, Chicago, IL USA; 5 Multimeric Biotherapeutics, Inc., La Jolla, CA USA

## Abstract

Donor-specific CD4^+^CD127^−^CD25^+^FOXP3^+^ regulatory T cells (AgTregs) have the potential to induce clinical transplant tolerance; however, their expansion *ex vivo* remains challenging. We optimized a novel expansion protocol to stimulate donor-specific Tregs using soluble 4-trimer CD40 ligand (CD40L)-activated donor B cells that expressed mature antigen-presenting cell markers. This avoided the use of CD40L-expressing stimulator cells that might otherwise result in potential cellular contamination. Purified allogeneic “recipient” CD4^+^CD25^+^ Tregs were stimulated on days 0 and 7 with expanded “donor” B cells in the presence of IL-2, TGFβ and sirolimus (SRL). Tregs were further amplified by polyclonal stimulation with anti-CD3/CD28 beads on day 14 without SRL, and harvested on day 21, with extrapolated fold expansion into the thousands. The expanded AgTregs maintained expression of classical Treg markers including demethylation of the Treg-specific demethylated region (CNS2) and also displayed constricted TcR repertoire. We observed AgTregs more potently inhibited MLR than polyclonally expanded Tregs and generated new Tregs in autologous responder cells (a measure of infectious tolerance). Thus, an optimized and more clinically applicable protocol for the expansion of donor-specific Tregs has been developed.

## Introduction

Organ and tissue transplantation currently rely on nonspecific immunosuppressive agents (IS) given life-long to prevent graft rejection. Although the introduction of new immunosuppressive agents, in particular calcineurin inhibitors (CNI), has resulted in a dramatic reduction in acute rejection rates following renal transplantation, these drugs have failed to prevent chronic allograft dysfunction (CAD). Moreover, these IS are associated with significant morbidity, particularly to the kidneys, and increased rates of infections, malignancy, diabetes, and hypertension. Therefore, since organs were first transplanted in humans, the elusive goal has been to establish donor-specific immunological tolerance, a state where a donated organ is accepted as “self,” eliminating the need for IS. To date the best clinical success at achieving tolerance has been through the combined use of hematopoietic stem cells (HSC) and solid organ transplantation^1–3^. However, the long term safety, efficacy, and broad applicability of approaches using therapeutic transfer of donor HSC is not yet known.

Regulatory CD4^+^CD25^+^FOXP3^+^ T cells (Tregs) have been shown to be elevated in tolerant human transplant recipients, and polyclonally expanded Tregs have been used to delay allograft rejection in experimental animals^4,5^ including humanized mice^6^, despite contrary results recently obtained in a heart transplant model in non-human primates^7^. As such, a number of centers, including our own, are beginning to explore the use of autologous regulatory T cells clinically to induce transplant tolerance^8,9^.

Polyclonally expanded Tregs have also been shown to have possible benefit in human GVHD^10–12^ and diabetes^13^. At our center, we have perfected large scale isolation and polyclonal expansion of good manufacturing practice (GMP)-grade autologous Tregs and successfully completed a phase 1 safety trial in living donor kidney transplant patients with no study-related adverse events^9^. Additionally, these patients developed increased numbers of circulating Tregs post-infusion. Based on this, we are poised to pursue a phase II efficacy trial.

Compared to polyclonally-expanded Tregs, donor-specific Tregs have the potential advantage of being more potent and specifically targeted to control alloimmune responses^14^. Therefore, a number of groups have developed Treg expansion protocols with restricted T cell receptor (TCR) repertoire recognizing only donor antigens and have shown such Tregs to delay graft rejection in rodent models of solid organ transplantation^14–17^. In this manuscript we have tested the hypothesis whether we can generate and expand in culture potent antigen-specific Tregs that can potentially be utilized clinically for tolerance induction.

A critical reagent to generate these alloantigen-specific Tregs is the donor antigen-presenting cells (APC). The source of these APC has been peripheral blood mononuclear cells (PBMC) alone^18^ or, in combination with FACS sorting^19^, dendritic cells^20^, and B cells^14,21^. Of these methods, activated B cells have been used most often. However, current published protocols rely on CD40L-expressing feeder cells for B cell activation and expansion, which have raised issues about their suitability for use in patients. Recently developed 4-trimer soluble CD40L circumvents the need for CD40L-expressing feeder cells to produce the adequate activation and expansion of B cells for use as APCs.

This study examines the effect of a 4-trimer soluble form of CD40L (UltraCD40L) on the expansion and activation of B cells, demonstrates generation of donor-specific Tregs using these activated B cells as APCs, and reports the ability of these Tregs to inhibit “recipient” anti-“donor” responses and preferentially induce the generation of new Tregs from “recipient” naïve CD4^+^ cells. Thus, an optimized and more clinically applicable protocol for the expansion of Tregs has been developed. It should be stressed that this report is not to provide a side-by-side comparison against other protocols being utilized elsewhere, but rather to describe the development of a Treg expansion protocol that does not require complicated starting cell purifications or CD40L-expressing feeder cells.

## Results

### The cellular reactants

In the proposed transplant situation, recipient Tregs are expanded against the organ or tissue donor and infused into the recipient after the transplant. Logistically, this can be achieved by pre-transplant non-mobilized leukophoresis of the recipient and cryopreservation of the product. Simultaneously, donor cells from either peripheral blood of a living donor or spleen cells of a deceased donor are obtained and the B cells are expanded and then cryopreserved, if required. Twenty-one days before the expected Treg infusion, the recipient leukophoresis product is thawed and the CD4^+^CD25^+^ cells are isolated and stimulated with expanded and irradiated donor B cells. Towards this, as a proof of principle, the following novel *ex vivo* expansion protocol was developed and optimized using peripheral blood mononuclear cells (PBMC) from two normal volunteers, henceforth denoting the individual from whom Tregs were isolated as “recipient” and the allogeneic B cells donor as “donor”.

### Generation of Activated B cells from “donor” PBMC in culture

Previous reports have demonstrated that B cells can be expanded using activation by cells expressing membrane CD40L plus IL-4 along with T-cell inhibition by CsA in culture^21^. In the current study, a similar system was developed that used a cell-free source of CD40L, namely UltraCD40L (a 4-trimer form of CD40L produced as a fusion between the body of surfactant protein D (SPD) as a scaffold and the extracellular domain of CD40L, also referred to as SPD-CD40L). The 4-trimer structure of UltraCD40L allows for better clustering of the CD40 molecules on B cells and thus increased expansion^22,23^. The UltraCD40L was produced by transfected CHO cells cultured in RPMI-1640 medium supplemented with 10% normal AB^−^ serum. The concentration of UltraCD40L in the culture supernatant ranges from 2.5–6 μg/ml and was used at 20% vol/vol for B cell expansion.

**Figure 1:**
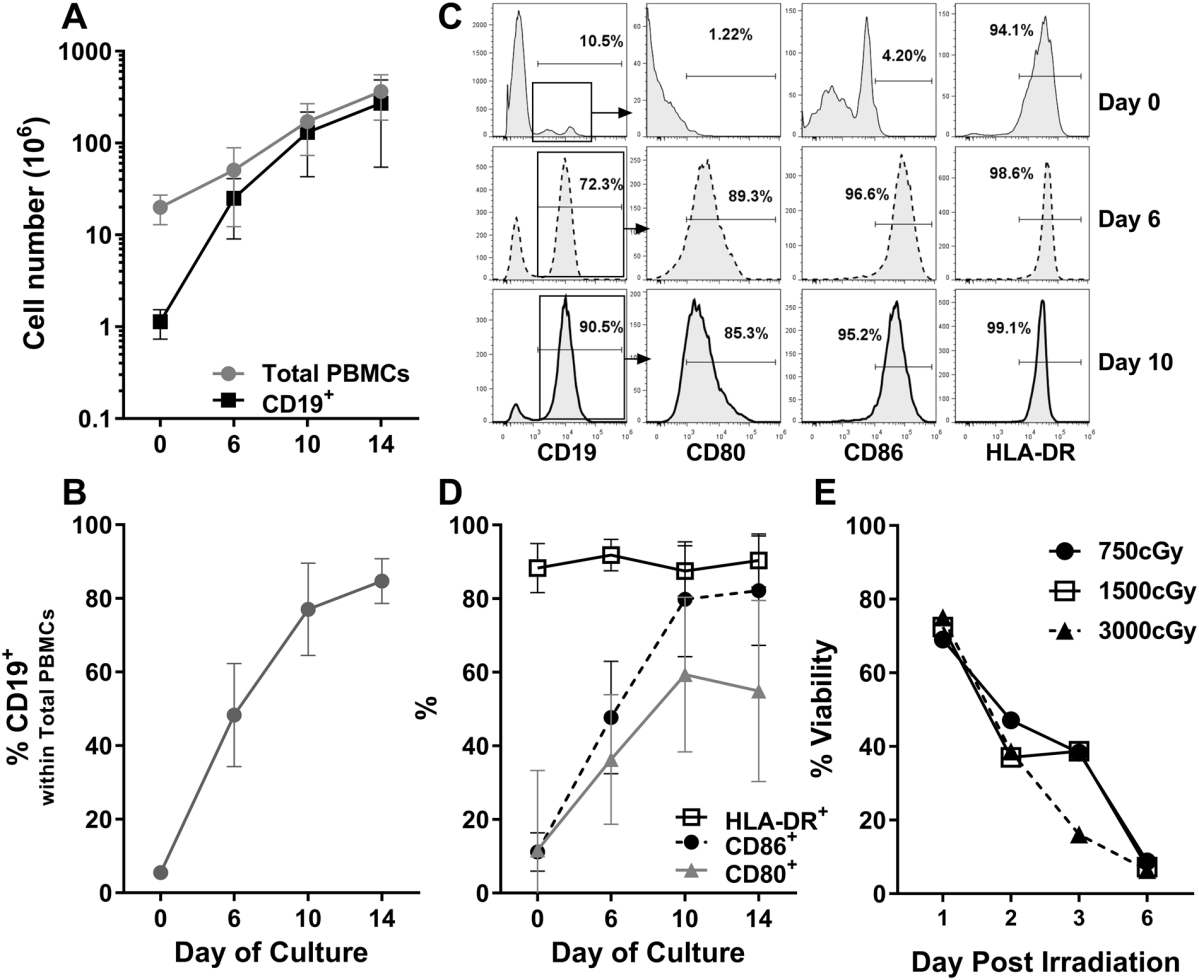
Robust expansion of “donor” B cells to be used as stimulators using ultraCD40L (n = 8). Peripheral blood was obtained from healthy volunteers and PBMC were isolated using Ficoll centrifugation. Either PBMC or CD19^+^ B cells purified using Miltenyi microbeads were cultured for 14 days in B cell culture media containing 20% ultraCD40L and 40ng/mL IL-4. Cyclosporine at 400ng/mL concentration was also included with PBMC cultures to prevent the growth of T cells. Cells were counted and flow cytometric analysis were performed on indicated days. (**A**) Absolute number of cells in the culture (mean ± SD); (**B**) Percentage of CD19^+^ B cells in the cultures where the starting cells were total PBMC; (**C**) A representative flow cytometry analysis showing the gating strategy for the classic B cell marker (CD19) and activation markers (CD80, CD86, and HLA-DR) in CD19^+^ gated cells. (**D**) The percentages CD19^+^ cells that expressed the activation markers on indicated days. Please note the robust expansion of B cells displaying high expression of HLA-DR, CD80 and CD86, hallmark of effective antigen presenting cells. (**E**) The expanded B cells that were to be used as stimulator cells were irradiated at indicated centi-Grey and cultured in 15% Human AB serum supplemented RPMI-1640 medium (15% HAB Medium). No viable B cells could be observed beyond 1 week of the culture, indicating that no B cell contamination was to be expected in the final expanded Treg product (of Fig. 2).

“Donor” PBMC were cultured under stimulation with UltraCD40L and 40 ng/mL IL-4 in the presence of 400ng/ml CsA to prevent the growth of T cells. We monitored the number of total cells and CD19^+^ cells in the culture to evaluate B cell growth. There was ~10-fold increase in the absolute number of cells after 14 days in culture when PBMC was the starting population, as opposed to a ~100-fold expansion if the initiating culture was CD19^+^ purified by immunomagnetic beads (Fig. 1A). The phenotypic nature of the expanded cells were monitored by 5-color flow cytometric analyses performed on a Beckman-Coulter FC500 flow cytometer as previously described^9,24,25^. This growth in the PBMC cultures was due to the amplification of the number and proportion of CD19^+^ B cells (Fig. 1B). The B cell cultures also showed an increased percentage of cells that expressed CD86 and CD80 (Fig. 1C and D) with the majority expressing HLA-DR throughout the culture. Thus, these expanded “donor” B cells demonstrated the phenotype of mature APCs. A detailed monitoring with more frequent flow cytometric analysis revealed that the maximal cell growth with optimum costimulatory molecule expression occurred on day 11-12, and therefore, subsequent B cell cultures were harvested at that period (data not shown). Since we obtained equally pure and activated B cells from cultures initiated from total PBMCs or purified CD19^+^ cells, for ease of clinical translation all further B cells expansions were performed from total PBMCs. The expanded B cells could be utilized either freshly or after cryopreservation with equivalent stimulatory capabilities (data not shown).

### Expansion of Purified Tregs with stimulation by activated B cells

**Figure 2:**
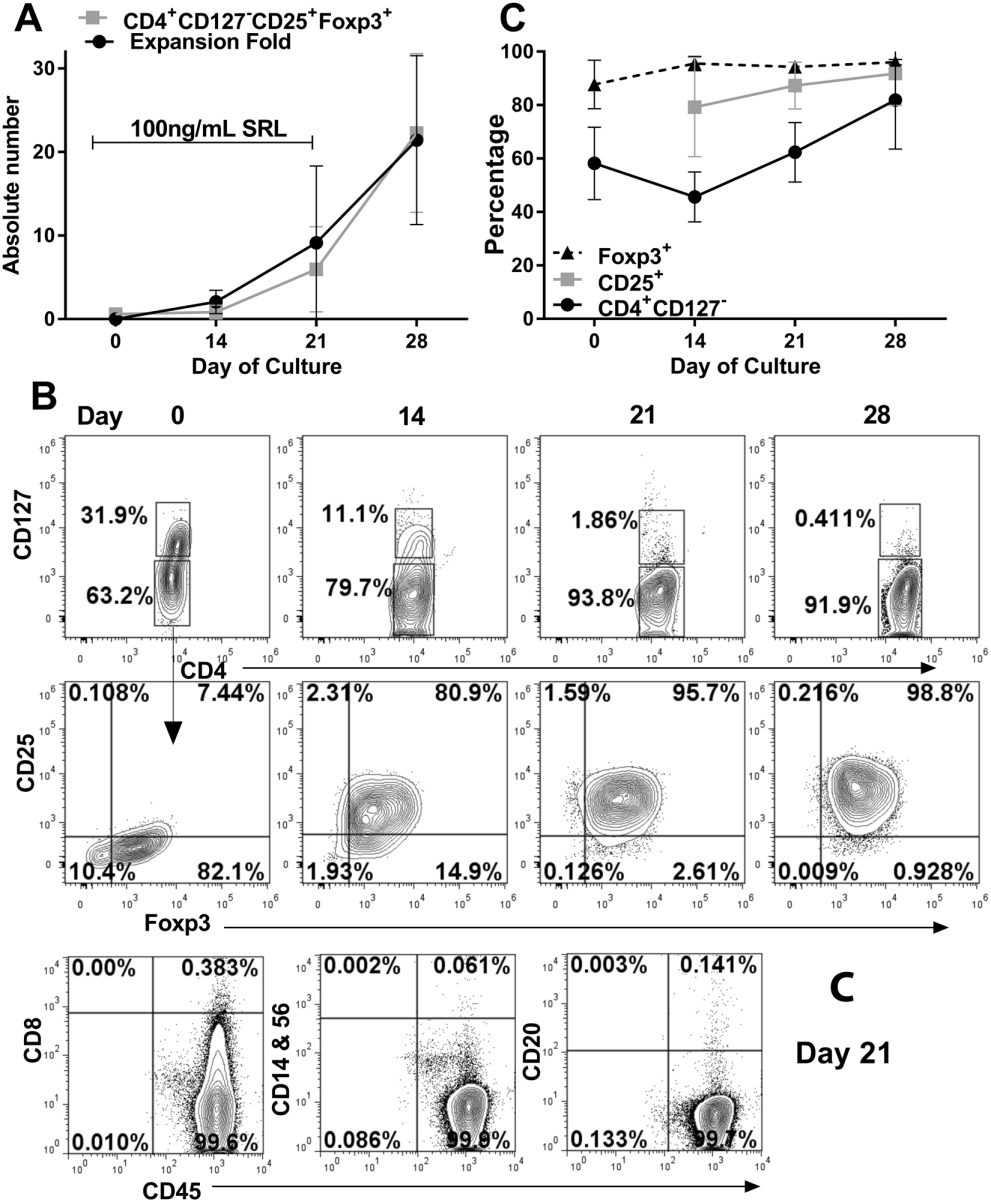
Expansion of AgTregs (n = 6). 1 × 10^6^ CD4^+^CD25^+^CD127^low^ cells were purified from healthy volunteers and stimulated with equal number of “donor” irradiated B cells that were expanded as in Fig. 1 and had one HLA-DR in common with the Tregs. Rapamycin at 100ng/mL was also included during the first 21 days. Tregs were restimulated the “donor” B cells at weekly intervals. (**A**) Tregs expanded ~20 fold during the 28 days in culture with the rapid expansion occurring after the removal of SRL on day 21. (**B**) Representative data of the flow cytometric scheme and analysis that demonstrated the gradual disappearance of residual CD127^dim^ cells with culture progression (top row), and robust expression of CD25 and FOXP3, the hallmark of Tregs (bottom row). [Note: Because of the flow cytometric configuration available in the laboratory, the CD25^+^ could not be accurately assessed on day 0 due to steric hindrance of the detection antibody by the selection antibody-beads.]. (**C**) Residual contamination by non-Treg subsets of cells were also monitored. No contamination with CD8^+^ T cells, CD14^+^ monocytes, CD56^+^ NK cells or CD20^+^ B cells was observed.

Next, we determined if purified CD4^+^CD25^+^ cells could be expanded using these activated B cells. The Tregs were isolated from “recipient” PBMC using Miltenyi Treg isolation kit II and were then cultured in 24-well culture plates with irradiated (3,000 cGy) “donor” B cells that had been expanded and activated in culture as above at a ratio of 1:1. Culture medium consisted of RPMI-1640 supplemented with 15% human AB serum, 1,000 IU/mL IL-2 and 1ng/ml TGF-β. 100 nM sirolimus was added to cultures except for the final 7 days. In terms of absolute number of cells, the Tregs proliferated ~20-fold during 28 days in culture, with robust proliferation occurring after SRL was removed from culture on day 21 (Fig. 2A). However, since <5% of the purified CD4^+^CD25^+^ are expected to be alloreactive and even lower percentages could be specific to the particular stimulator, this would represent an expansion from 400 to >1,000 fold.

Phenotypically, the purified Tregs on day 0 prior to culture, were >95% CD4^+^ and >85% FOXP3^+^. A minor population of conventional T cells staining CD127^dim^ was present in the purified product. However, it gradually disappeared with the final product becoming virtually devoid of CD127^+^ cells (Fig. 2B, top row). Because of the flow cytometric configuration available in the laboratory at the time, the CD25^+^ population could not be assessed on day 0 for the starting product, as the selection antibody sterically hindered the detection antibody. However, there was a progressive enrichment with >90% being CD25^+^FOXP3^+^ cells at the end of the culture period (Fig. 2B, bottom right). Thus, >80% of the cells were CD4^+^CD127^−^CD25^+^FOXP3^+^ Tregs in the final product (Fig. 2C). Henceforth, these are designated as antigen-specific Tregs (AgTregs).

To test if any of the “donor” irradiated B cells still remained as a contaminant in the final Treg product, two approaches were utilized: (i) Expanded B cells were irradiated at varying cGrey and cultured in 15% HAB medium; no viable B cells could be detected beyond 1 week of the culture at any of the irradiation doses (Fig. 1E). This indicated that no B cell contamination was to be expected in the expanded Tregs. (ii) Expanded Treg product was monitored for any residual B cells; no contamination with CD20^+^ B cells was observed. Similarly, no CD14^+^ monocytes, CD56^+^ NK cells or CD8^+^ T cells were also detected. A representative analysis on a 21 day culture is shown in Fig. 2C.

### Expanded AgTregs demonstrate potent “donor”-specific MLR inhibition

**Figure 3:**
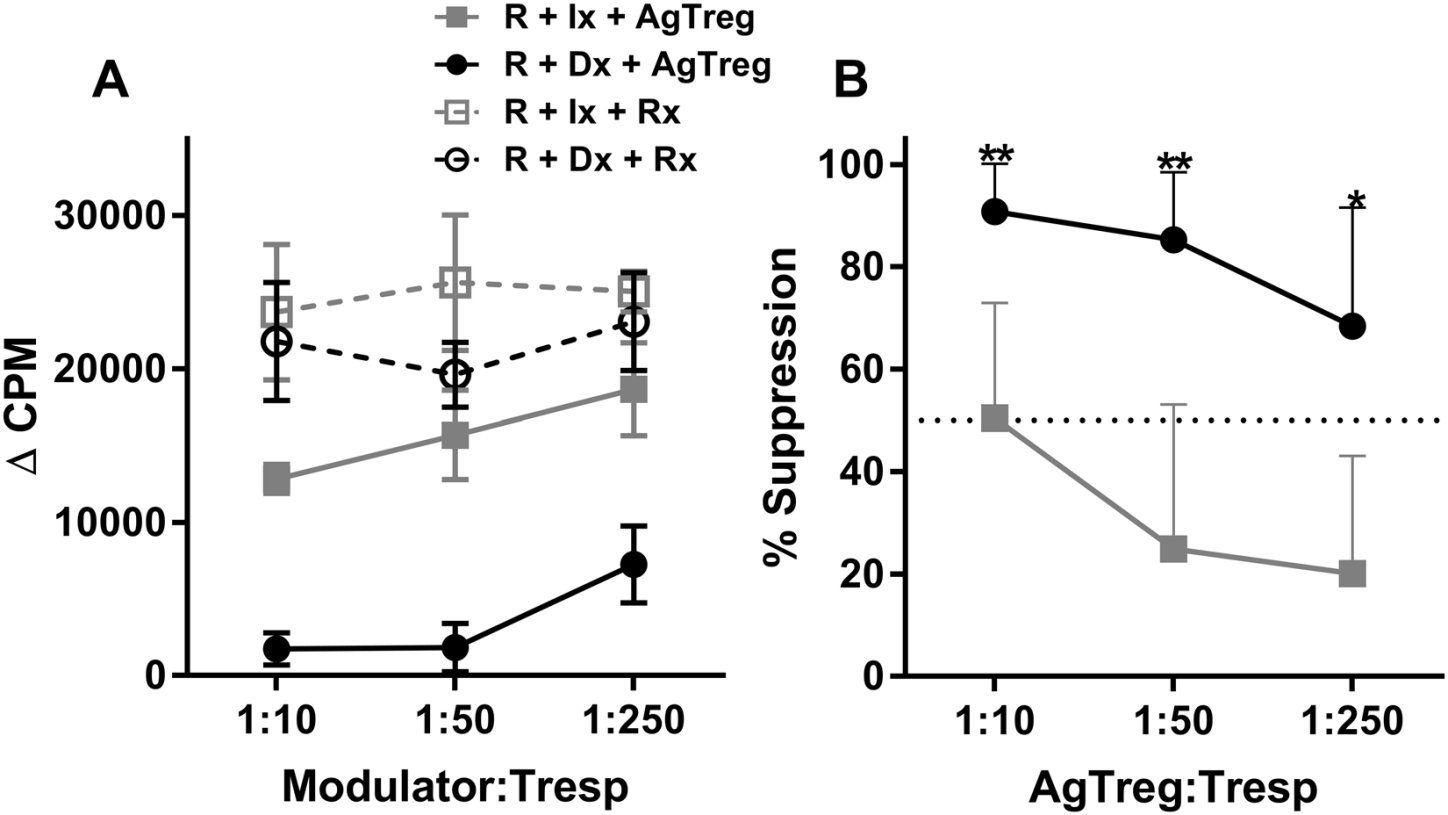
Specificity and Potency of AgTregs. On Day 28, AgTregs were harvested and were used as modulators in mixed lymphocyte reaction of autologous PBMC stimulated with irradiated PBMCs from either the donor used for expanding the Tregs or an irrelevant third party. Additional responder PBMC was used as control modulators. Thus the combinations were [Recipient responder PBMC + donor irradiated stimulator + control = (R + Dx + Rx)], [Recipient responder PBMC + donor irradiated stimulator + AgTregs = (R + Dx + AgTregs)], [Recipient responder PBMC + irrelevant irradiated stimulator + control = (R + Ix + Rx)] and [Recipient responder PBMC + irrelevant irradiated stimulator + AgTregs = (R + Ix + AgTregs)]. After 7 days, a standard thymidine incorporation assay was performed. (**A**) Representative experiment showing the delta counts per minute (ΔCPM ± SD) values with the various modulators at indicated modulator: T responder ratios. (**B**) Data are shown as mean ± SD percentage of suppression that were calculated for each individual experiment (n = 9) using the formula shown in the text. Tregs demonstrated potent MLR inhibition against the specific donor stimulator with minimal inhibition against the third party, thus showing the antigen specificity of the inhibition. *p < 0.05; **p < 0.01.

To examine the functional potency and specificity of expanded Tregs, MLR inhibition assays were performed. “Recipient” responder PBMC autologous to the Tregs were stimulated with irradiated PBMC from either the “donor” stimulator used in the generation of Tregs or a third party irrelevant individual in presence of either expanded AgTregs or irradiated “recipient” cells as modulator controls. The back-response CPM given by the AgTregs or control modulators at the tested doses [as assessed in (Rx + Dx + AgTregs) combinations] were subtracted from the (R + Dx + AgTregs) experimental cultures to obtain the delta CPM (Δ CPM), and the percentage of inhibition was calculated. Figure 3A shows the results from a representative experiment as Δ CPM obtained in the cultures with AgTreg or control modulators at indicated ratios with “recipient” PBMC responders. When the percentage of inhibition by each ratio of AgTregs versus the control modulator in each experiment was calculated, a dose dependent and potent inhibition was observed (Fig. 3B). This inhibition was significantly higher in the MLR against the specific donor stimulator than against irrelevant third party stimulator, thus demonstrating the antigen specificity of the expanded AgTregs.

### Cultured antigen-specific Tregs generate new Tregs by infectious tolerance

**Figure 4:**
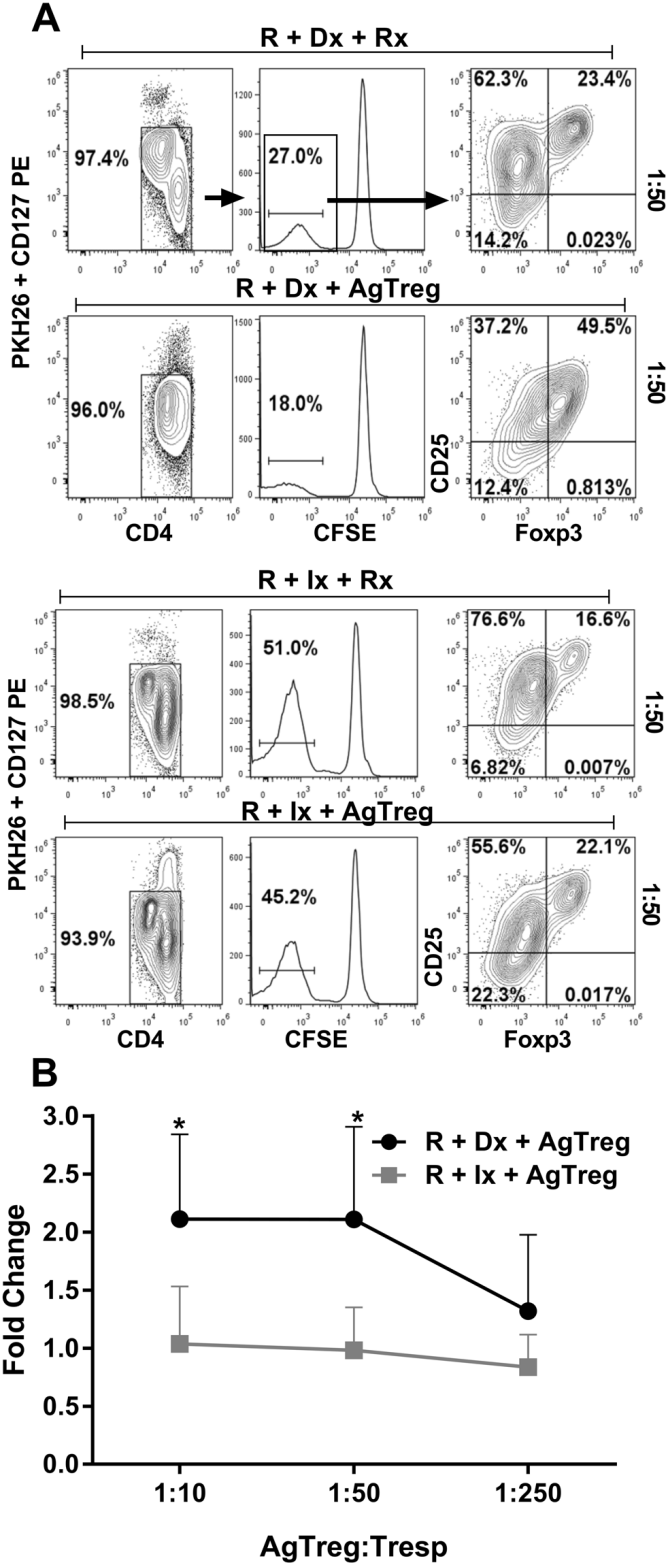
Infectious Generation of new Tregs in responders by expanded AgTregs. Responder PBMCs (R) were labeled with CFSE; and stimulator Dx and Ix as well as the modulators (Rx and Tregs) were labeled with PKH26 prior to assay performance. The responders were cultured with equal number of irradiated stimulators in presence of indicated ratios of the modulators. After 7 days flow cytometric analysis was performed with monoclonal antibodies CD127-PE, CD4-ECD, CD25-PC7 and FOXP3-PC5. (**A**) *Flow cytometric gating strategy*: The CD4 cells that were negative for PKH26 and CD127-PE and then those that diluted the CFSE were sequentially gated and analyzed for CD25 and FOXP3 expressions. Thus, the cells of interest were CD4^+^CD127^−^CD25^+^FOXP3^+^ Tregs in the CFSE diluted proliferating responders. A representative experiment with “donor” and third party stimulators at 1:50 modulator: T responder ratio is shown. (**B**) The percentages of CD4^+^CD127^−^CD25^+^FOXP3^+^ Tregs obtained with the AgTreg modulators were divided by those obtained with the Rx control modulators to calculate the fold change. Thus, in the example in Fig. 4A left, the fold change was 49.5 ÷ 23.4 = 2.12. This fold change in Tregs was plotted for the donor-specific and third party responses at indicated AgTreg: T responder ratios for each experiment. The data are shown as mean ± SD fold change from n = 5 experiments. *p < 0.05. There was an amplification of Tregs that were newly generated in the proliferating fraction of autologous responders by the expanded AgTregs in a “donor” specific manner.

One potential advantageous characteristic for maintaining a lasting immune tolerance by infused Tregs is that they can cause the generation of new Tregs in the recipients which is termed “infectious tolerance”^26^. The Treg-MLR assay developed in the laboratory is an *in vitro* correlate of this, and we employed it to assess the ability of expanded AgTregs in generating new Tregs from naïve autologous responder cells. We utilized total PBMCs and not Treg depleted PBMCs as responder cells because, clinically, patients that receive Treg infusion will have native Tregs present and therefore the overall effect/ability of infused Tregs to generate new Tregs within recipients is better assessed with total PBMCs. Responder PBMC labelled with CFSE were stimulated with either PKH26-labelled and donor irradiated PBMC or third party irrelevant irradiated PBMC in the presence of PKH26-labelled modulator cells (AgTregs or additional responder PBMC as controls) at varying concentrations. The percentages of CD4^+^CD127^−^CD25^High^FOXP3^+^ cells that were generated in CFSE diluted proliferating responder cells were determined by flow cytometry on day 7 and analyzed after gating out all PKH^+^ modulators and residual stimulators as well as CD127-PE positive effector cells (Fig. 4A).

When autologous PBMC served as the modulator cells for either “donor”-specific (R + Dx + Rx) or third party indifferent (R + Ix + Rx) reactions, the percentage of CD4^+^CD127^−^CD25^High^FOXP3^+^ Tregs in the proliferating responders remained constant at around 20% at all modulator doses tested (Fig. 4A). However, there was a two-fold (200%) increase in the proportion CD4^+^CD127^−^CD25^High^FOXP3^+^ cells among proliferating responders when AgTregs were added as modulator cells into the “donor”-specific (R + Dx + AgTreg) cultures at 1:10 and 1:50 Treg: T-responder ratios (Fig. 4B, top line). In contrast, no such increase in the Tregs newly generated in the responders was observed in the third party specific (R + Ix + AgTreg) cultures (Fig. 4B, bottom line). These results demonstrate that AgTregs are capable of infectiously generating new Tregs in autologous naïve responder cells.

### Further optimization of antigen-specific Treg expansion protocol for the GMP

**Figure 5:**
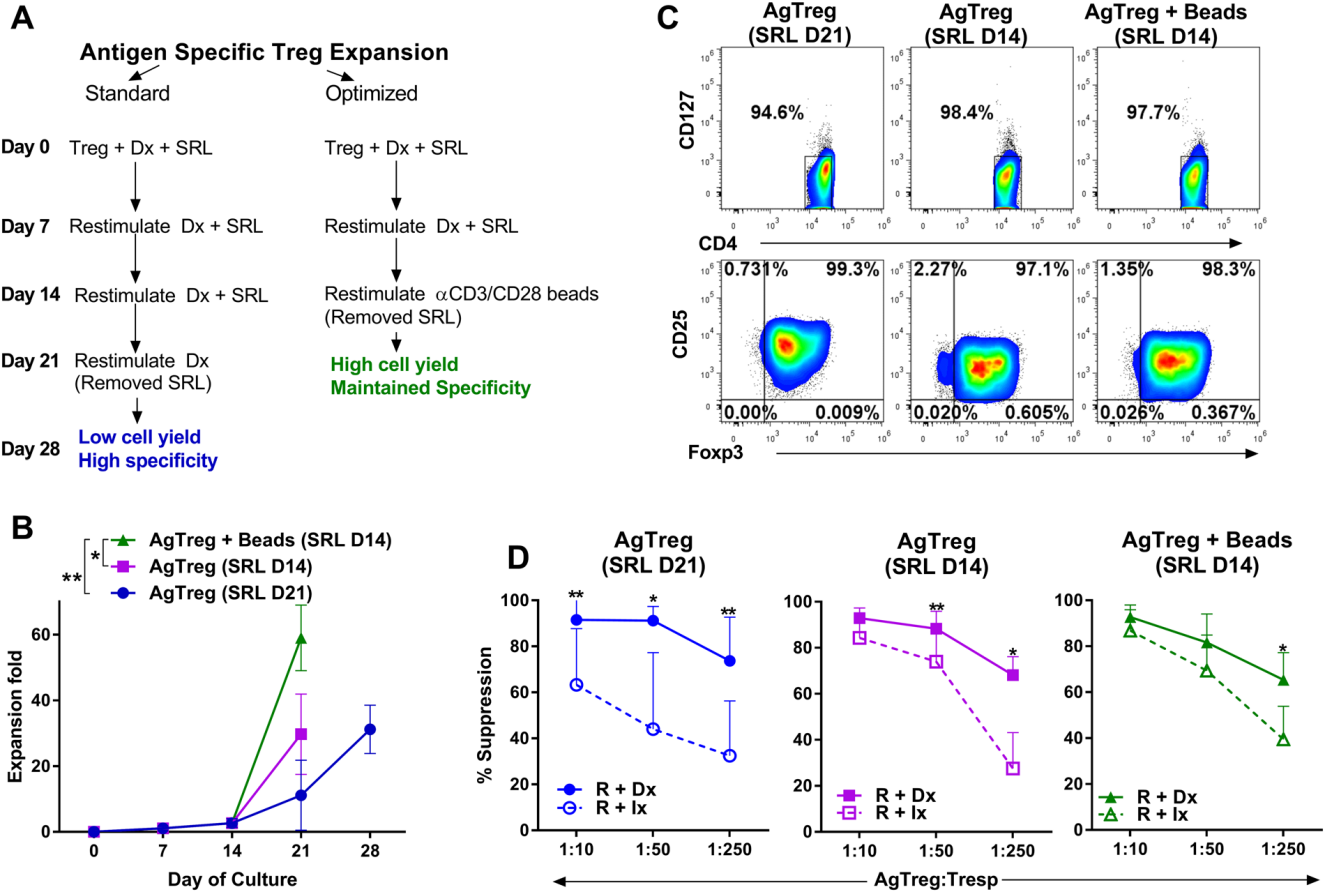
Optimization of AgTreg Expansion with Polyclonal Re-stimulation. To improve expansion potential, the standard expansion protocol was modified with antigen specific B cell stimulation on days 0 and 7 followed by polyclonal stimulation with anti-CD3/CD28-beads on day 14. (**A**) Flow chart showing the method used to develop the technology (standard) vs. the method modified for transfer to GMP (optimized). (**B**) Fold expansion (Mean ± SD) with the optimized method of anti-CD3/CD28-bead restimulation and removal of SRL on day 14 (green line with triangular markers), with antigen-specific restimulation and removal of SRL on day 14 (purple line with square markers) and standard method (blue line with round markers). n = 7. (**C**) Representative experiment showing the phenotypic profile in terms of CD127, CD25 and FOXP3 expressions in CD4^+^ gated cells. (**D**) Comparison of “donor”-specific vs non-specific suppression by AgTregs expanded using the three methods and MLR inhibition performed as described in Fig. 3. n = 6, *p < 0.05 **p < 0.01. The method optimized for transfer to the GMP facility demonstrated superior expansion without the loss of antigen specificity.

We recognize that although our AgTregs are highly potent and specific, an expansion protocol with duration of a month will be costly and time consuming for clinical applications. We addressed this concern by asking if amplified expansion without the loss of antigen specificity could be obtained by polyclonal restimulation after two rounds of antigen specific stimulations. Purified CD4^+^CD25^+^ cells were stimulated “donor”-specifically on days 0 and 7, and then polyclonally expanded with MACS GMP ExpAct Treg beads at 1:1 ratio on day 14 without SRL. The cultures were harvested on day 21 and compared against the standardized protocol described above. We also included another comparator wherein the third stimulation on day 14 was also antigen-specific and in the absence of SRL. When analyzed on day 21, all the three cultures (Fig. 5A) showed equivalent proportion of cells that were CD4^+^ and CD127^−^ as well as CD25^+^ and FOXP3^+^ (Fig. 5B), proving polyclonal restimulation does not negatively affect Treg phenotype. However, there was a two-fold increase in the absolute number of Tregs when polyclonal restimulation was used as compared to antigen-specific stimulation throughout, both in the absence of SRL during the final 7-day culture period (Fig. 5C). When assayed functionally in MLR inhibition assays on day 21, all three cultures demonstrated equivalently potent inhibition against the specific “donor” stimulator used in expanding the Tregs (Fig. 5D, top solid lines). However, there were subtle differences in the inhibition against the third party irrelevant stimulated MLRs (Fig. 5D, bottom dashed lines); the cultures from which SRL was withdrawn on day 14 (middle and right) had higher inhibition at 1:10 and 1:50 Treg: responder ratios, when compared to the culture with SRL present throughout the 21 day culture (left). Importantly, the inhibition was “donor”-specific in all cultures at higher and more clinically relevant Treg: responder ratios (>1:250). These results demonstrate that amplified expansion without the loss of antigen specificity can be obtained by polyclonal restimulation on day 14 subsequent to antigen specific stimulations on days 0 and 7.

### Comparison of antigen-specific versus polyclonally expanded Tregs

**Figure 6:**
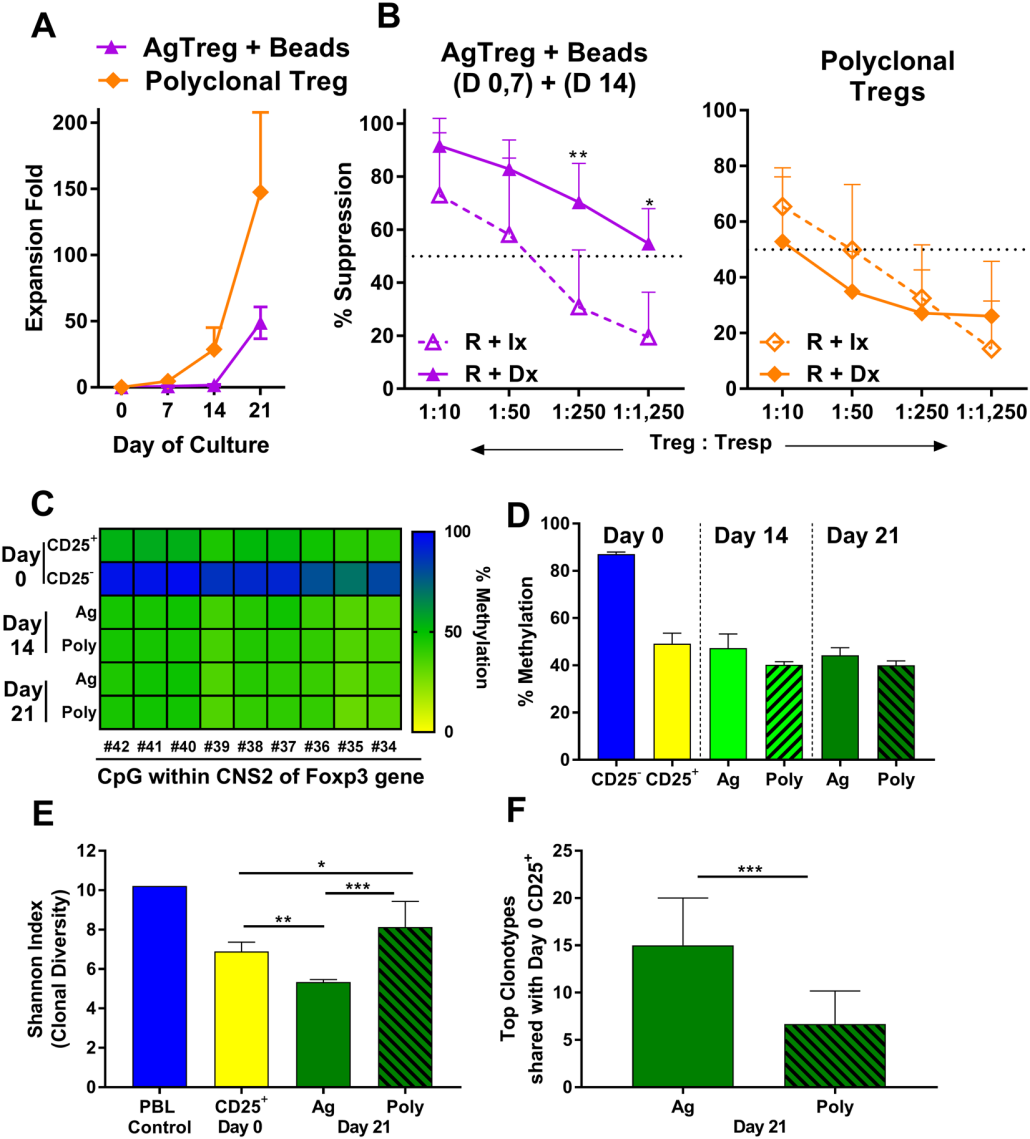
Superiority of Antigen Specific Tregs versus polyclonal Tregs. Tregs were expanded for 21 days from the same individual by (i) antigen-specific stimulations on days 0 and 7 plus polyclonal stimulations with anti-CD3/CD28-beads on day 14 (triangle markers), and (ii) polyclonal stimulations with anti-CD3/CD28-beads on days 0, 7 and 14 (diamond markers). SRL was withdrawn from the cultures on day 14. (**A**) Fold expansion with the two methods of Treg expansions (Mean ± SD; n = 7). (**B**) Comparison of “donor”-specific vs non-specific suppression by Tregs expanded using the two methods and MLR inhibition performed as described in Fig. 3. n = 7 (please note that for the dilution 1: 1,250 the n = 4 only). The dotted line represents 50% inhibition. *p < 0.05. (**C**) Heat map of methylation analysis of sub-fractions of cells from a representative experiment. (**D**) Methylation status of freshly isolated effector cells and Tregs (Day 0) as well as Tregs expanded either antigen-specifically or polyclonally on days 14 and 21. Cells assayed without purification of Tregs and the results were plotted without correction for X-linked inactivation^38^, even though all Tregs were generated from females (n = 3). (**E**) Mean ± SD clonal diversity observed in 21 day cultures of AgTregs (n = 3) and polyclonally expanded Tregs (n = 3) versus Day 0 CD25^+^ starting population of cells (in duplicate). (**F**) The top clonotypes shared by 21 day cultures of AgTregs and polyclonally expanded Tregs (n = 3) versus Day 0 CD25 + starting population of cells. P = * < 0.05, ** < 0.01, *** < 0.001. As expected, the fold expansion by “donor”-specific stimulations was lower. The AgTregs demonstrated higher and specific inhibition at all Treg: T responder ratios. The Tregs demonstrated stable demethylated state of the FOXP3 gene. The AgTregs showed a slight shrinkage in the clonal diversity (as to be expected) but had higher top clonotypes in common with the starting cells than polyclonally expanded Tregs.

We recently developed the technology for the expansion of Tregs polyclonally and completed a phase I safety trial in kidney transplant recipients with these polyclonally expanded autologous regulatory T cells^9^. Therefore, it was of interest to make a direct comparison of the characteristics of polyclonal versus AgTregs. We also purified the Tregs by first depleting non-CD4 cells and then positively selecting CD25^+^ cells using the reagents and CliniMACS system that would be used for clinical expansion. Tregs were expanded from the same individuals using the optimized AgTreg and polyclonal Treg expansions, i.e. (i) antigen-specific stimulation on days 0 and 7 followed by restimulation on day 14 with anti-CD3/CD28 beads, and (ii) polyclonal stimulation with anti-CD3/CD28 beads on days 0 and 7. Both the cultures were in the absence of SRL from day 14 onwards and harvested on day 21. As expected, the fold expansion was highest with polyclonal stimulations (Fig. 6A). Phenotypically, both cultures demonstrated equivalent characteristics and similar to those shown in Figs 2 and 4. However, when tested as modulators in MLRs, the polyclonally expanded Tregs displayed less inhibition of MLR compared to AgTregs which inhibited the responses in an antigen-specific manner, especially at the higher Treg: T-responder ratios (Fig. 6B). For instance, ~75% inhibition was demonstrated by AgTregs versus ~35% by polyclonally expanded Tregs at Treg: T responder ratio of 1:250. In another series of experiments in which head-to-head comparisons were not made, polyclonally expanded Tregs showed lower dose-dependent inhibition also (n = 9; not shown). These results indicated that antigen-specific Tregs are more potent and specifically targeted.

### Expanded Tregs demonstrated stable FOXP3 demethylation profile

The demethylation of a conserved region in the first intron of the Foxp3 gene Treg-specific demethylated region (TSDR) has been demonstrated as the most reliable criterion for identification of Tregs^27,28^. Therefore, the methylation / demethylation status of the expanded Tregs was compared against those of a number of cell subsets by bisulfite conversion, and pyrosequencing of isolated DNA through the use of a commercially available vendor services (EpigenDx). As can be seen in Fig. 6C and D, freshly isolated CD25^−^ effector T cells, the negative controls had >90% cells methylated and the starting population of CD25^+^ cells (Day 0) had ~50% cells methylated. The polyclonally expanded Tregs retained similar methylation / demethylation status throughout the culture. Similarly, the AgTregs also demonstrated equivalent TSDR despite the analyzed samples were the total Treg culture, without depletion of residual contaminating stimulator DNA (this was done as the total cellular product would be infused into the patient). Please also note that no correction for X-linked inactivation^29^ was made when the results were plotted. These results showed that the expanded Tregs had stable TSDR.

### Expanded AgTregs have restricted but sufficiently broad TcR repertoire usage

A diverse T cell repertoire has been found to be essential to maintain an adequate level of recognition and response to environmental antigens^30^ and this is true even for regulatory T cells controlling responses to the wide range of antigens^31^. Since alloreactivity encountered in a transplant situation has an array of antigens acting as immune targets, a wide range of clonal diversity is expected. Thus, a TcR repertoire analysis (curtesy of ArcherDx, Boulder, CO), revealed that there was sufficient breadth, albeit with significant shrinkage in the TCR diversity in the AgTregs cultured for 21 days from the Day 0 CD25^+^ starting population of cells (Fig. 6E). This was in contrast to an expansion in clonal diversity in the 21-day polyclonally expanded Tregs (i.e. a more polyclonal repertoire); this was likely due to uncovering of low frequency clones within the Treg product that were below the detection level in the starting cells and not by the generation of new clones as expansion was an *ex vivo* procedure. However, when the top clonotypes that expanded in culture were assessed, the AgTregs had more clonotypes in common with the starting cells than the polyclonal Tregs (Fig. 6F) thus indicating that the more abundant clones in the CD25^+^ product were the ones that expanded in AgTreg cultures. Overall, Treg receptor diversity was maintained post expansion in the polyclonal product with the expected shrinkage in the AgTregs.

## Discussion

This study describes the development of the technology for effective isolation and expansion of allospecific regulatory T cells through the use of “donor” B cells that have been expanded by a novel clinically applicable method. Several reports^14,21^ describe the challenges in the generation of large numbers of antigen-specific Tregs for clinical use. The first step in the process is to produce large numbers of “donor” APC to stimulate and expand donor antigen-reactive Tregs. Although Tu *et al*. first reported roughly equal effectiveness of soluble CD40L compared with CD40L-transfected feeder cells expressing membrane CD40L to generate activated B cells, most protocols currently rely on the latter which may produce cellular contamination and limit clinical use. Using newly developed technology where 4 trimers of CD40L are linked together (UltraCD40L), large quantities of activated B cells can be expanded without feeder cells, thus making this approach more clinically applicable. This is one of the features that distinguishes this study from others, i.e. the use of cell-free, soluble 4-trimer CD40L (Ultra-CD40L) to expand “donor” B cells.

Our simplified B cell expansion protocol used in these experiments has thereby produced similar expansion and activation (Fig. 1) of B cells from PBMC compared to previous reports^14,21^. Whereas most protocols begin by isolating B cells from PBMC we found this step unnecessary and achieved equally robust expansion when starting from unfractionated PBMC. Under our culture conditions using CsA to suppress T cell growth along with B cell stimulation by UltraCD40L and IL-4, we produced highly immunogenic mature APC expressing CD86, CD80 and HLA-DR. This protocol thereby produced >200 million B cells starting with 20 mL of peripheral blood, i.e., ~100-fold expansion, indicating that clinical-scale production would be possible assuming 2–3 billion stimulator B cells would yield an effective number of antigen-specific Tregs.

The AgTregs produced in this study also confirm reports by others that activated B cells can be used to select (“donor”) antigen-specific Tregs^14,21^. In our final optimized protocol now poised to be transferred to our clinical GMP facility, the Tregs were stimulated “donor”-specifically on days 0 and 7 in presence of SRL and then polyclonally with anti-CD3/CD28 on day 14 without SRL, and the cells harvested on day 21. Another distinguishing feature of our protocol is that the starting population of cells is prepared using a less cumbersome method than that reported by Putnam *et al*.^14^ who used flow-sorting of the starting Treg cells (and CD40L-expressing K562 leukemia cells as stimulators). However, the amplification of Tregs is specifically favored in our cultures by the use of 100ng/ml SRL to prevent the expansion of effector T cells^32–39^, giving ~60 fold expansion in the total number of Tregs by this protocol. Since < 5% of the purified CD4^+^CD25^+^ are expected to be alloreactive and even lower percentages could be specific to the particular “donor”, this would represent a fold expansion into the thousands.

One of the critical questions that is relevant to immunomodulation therapy with Tregs is whether sufficient numbers of cells can be obtained through the expansion protocol. In a typical human there are ~1,500 lymphocytes per µl of peripheral blood and there are ~5 × 10^6^ µl (5 liters) of blood, and therefore ~7.5 × 10^9^ total lymphocytes. In our renal transplant clinical protocol, we reduce the peripheral lymphocyte count to ~150 per µl (~7.5 × 10^8^ total) or lower by the use of lymphodepleting agents such as Alemtuzumab. Under these circumstances, infusion of 10^7^–10^8^ *ex vivo* expanded donor-specific Tregs is easily achievable, i.e., ~1:2 ratio of Treg to T responder in recipient peripheral blood after Alemtuzumab has been metabolized. Of course, this calculation does not take into account the recipient’s other lymphoid compartments. However, since the Tregs are highly potent and specific immunoregulators (Figs 3 and 5) and can infectiously induce the generation of new Tregs, even lower proportions may possibly be tolerogenic.

The stability and potency of the AgTregs are very important considerations in Treg expansion protocols. Since our expanded Tregs demonstrate stable TSDR, it may be assumed that they may not show plasticity by turning into Th17 inflammatory cells^40,41^. Similarly, the expanded AgTregs also demonstrated a restricted yet sufficiently broad TcR repertoire expected of alloreactive regulatory T cells. Further, the Tregs produced in this study appears to be potent specific MLR inhibitors (Fig. 6). Please note that the lowest ratio of Treg: T responder used here was 1:10 as opposed to 1:1 by others and the highest 1:1,250 by us vs 1: 125 by others^14^ as we had reasoned that infusion(s) of AgTregs would achieve only higher ratios *in vivo*, and therefore, only equivalent ratios would provide clinically meaningful results. The higher potency of our Tregs, whether allo-specific or polyclonal, is possibly due to the use of SRL in the initial expansion culture period.

Another critical factor that determines successful *in vivo* tolerance induction is the maintenance of amplified Treg proportion and functions in the recipient. This can be achieved through prolonged survival of the infused Tregs, as observed by Chandran, *et al*. in the peripheral circulation in humans^42^, and by Zhang, *et al*. in the circulation and secondary lymphoid tissues of non-human primates^43^. Alternate and relevant mechanisms conferring lasting clinical tolerance may include better homing and functioning^44^ and infectious tolerance described by Waldman and co-workers as well as others^45–47^. To assess this potential of AgTregs to infectiously generate new Tregs from naïve cells in the recipient, we have utilized the “Treg-MLR,” an *in vitro* correlate of the *in vivo* phenomenon^24^. Using this assay, we have demonstrated that proliferating autologous PBMC preferentially develop new Tregs with “donor”-specific but not third party stimulation (Fig. 4). Previously, we had shown that the CD4^+^ cells that were newly generated in the Treg-MLR would in turn inhibited fresh MLRs again in an antigen-specific manner demonstrating their own specific inhibitory capabilities^48^. Similar infectious tolerance through Treg development has been indirectly observed in transplant recipients after infusion of Tregs or donor hematopoietic stem cells^1–3,21,24,25,49–52^. The expanded AgTregs thus have tremendous clinical implications particularly in the induction and maintenance of transplant tolerance where current IS has failed long-term.

To summarize, this study has examined the effect of a cell-free, 4-trimer form of soluble CD40L (UltraCD40L) on the expansion and activation of B cells which in turn function as effective stimulators of antigen-specific regulatory T cells. Some of the salient and distinguishing features of the present study are: (i) the use of soluble 4-trimer CD40L to expand “donor” B cells instead of CD40L-expressing feeder cells; (ii) simplified purification of the initial “recipient” Treg cell to be expanded; (iii) stimulation of the Tregs with expanded “donor” B cells on days 0 and 7, then with anti-CD3/CD28-beads on day 14 and product harvest on day 21; and finally (iv) use of SRL in the initial stimulation phase (days 0–14) to prevent the expansion of effector T cells and favor the amplification of Tregs. We are in the process of transferring the technology to our GMP facility, so that a phase I clinical trial can be initiated in the near future. Thus, we have verified our hypothesis that we can generate and expand in culture potent antigen-specific Tregs that can potentially be utilized clinically for tolerance induction.

## Methods

### Subjects

The subjects of the study were normal laboratory volunteers. Peripheral blood samples were obtained from them under a protocol following written informed consent approved and supervised by a Northwestern University Institutional Review Board. All procedures followed were in accordance with the ethical standards of the responsible committee on human experimentation (institutional and national) and with the Helsinki Declaration of 1975, as revised in 2008. The healthy volunteers were HLA-typed by the Northwestern HLA laboratory using molecular methods (reverse sequence specific oligonucleotide probe hybridization). In a typical experiment B cells were expanded using UltraCD40L and these “donor” cells were used to stimulate and expand purified CD4^+^ CD127^-^CD25^+^ cells (Tregs) from an allogeneic individual designated as “recipient” using the following protocols.

### Generation of B cells from “donor” PBMC

Peripheral blood mononuclear cells (PBMC) were isolated using Ficoll-Hypaque density centrifugation and cultured in RPMI media (Invitrogen, Carlsbad, CA) containing 15% heat-inactivated human AB serum (Sigma, St. Louis, MO), HEPES buffer (Invitrogen), Penicillin/streptomycin/glutamine (Invitrogen) with the addition of 40 ng/mL IL-4 (R&D Systems), 20% soluble 4-trimer CD40L (SPD-CD40L fusion protein, UltraCD40L; Multimeric Biotherapeutics, La Jolla, CA), and 400ng/ml cyclosporine-A (Novartis, NJ) at 37 °C with 5% CO_2_. Cells were cultured in T75 flasks (Corning, NY), counted every 3–4 days, and maintained at a cell density of 0.5–1 × 10^6^ cells/mL. After 14 days in culture, the expanded B cells were phenotypically characterized by flow cytometry and were irradiated prior to being used as stimulators in the expansion of Tregs. Henceforth the volunteer from whom B cells were expanded and used as stimulators is denoted as “donor”.

### Treg isolation and expansion

PBMC were isolated from healthy human donors (mis)matched at one HLA-DR locus to the corresponding B cell donors. Initially, CD4^+^CD127^−^CD25^+^ Tregs were isolated using Miltenyi Treg isolation kit II (Miltenyi Biotec, Auburn, CA) by first depletion of CD8, CD19, CD123 and CD127 positive cells followed by positive selection for CD25^+^ cells. Subsequently at the final optimized stages, Tregs were isolated by first depletion of CD8 and CD19 positive cells and then positive selection for CD25^+^ cells using GMP grade reagents. Isolated Tregs were then cultured in 24-well culture plates (Corning) for 21 or 28 days with irradiated (3,000 rads) B cells that had been expanded and activated in culture as above at a ratio of 1:1. Culture medium consisted of RPMI-1640, 15% human AB serum, penicillin/streptomycin/L-glutamine, HEPES, 1,000 IU/mL IL-2 (R&D Systems), and 1ng/ml TGF-β (R&D Systems). 100 nM sirolimus (SRL; Pfizer, NY) was added to cultures except for the final 7 days. Initially, the cultures were restimulated on days 7, 14, and 21 with the irradiated B cells at a 1:1 ratio. In the final optimized protocol, the Tregs were stimulated with irradiated “donor” B cells at a 1:1 ratio on days 0 and 7 in presence of SRL and restimulated polyclonally with MACS GMP ExpAct Treg beads (Miltenyi Biotec) at 1:1 ratio on day 14 in the absence of SRL; the cells were harvested on day 21. For polyclonal expansion of Tregs isolated CD4^+^CD25^+^ cells were stimulated with MACS GMP ExpAct Treg beads at 1:4 ratio on days 0 and 1:1 on day 7 in the presence of SRL and on day 14 in absence of SRL; the cells were harvested on day 21. Tregs were cultured at a cell density of 1 × 10^6^ cells/mL with flow cytometric phenotyping performed at weekly intervals. Henceforth, the volunteer from whom Tregs are expanded is denoted as “recipient”.

### Flow Cytometry

For “donor” B cell expansion experiments, flow cytometry was performed on cultured PBMC on indicated days using antibodies against CD19-PC7, CD80-PE, CD86-PC5, HLA-DR-ECD (all from Beckman-Coulter, Miami, FL). To phenotype Tregs, antibodies against CD4-FITC, CD127-PE, CD3-ECD, CD25-PC7 (all from Beckman-Coulter) and FOXP3-PC5 (eBioscience, San Diego, CA) were used on days 0, 14 and 21, (also on day 28 in longer-term cultures). All detection was performed on a Beckman-Coulter FC500 flow cytometer as previously described^9,24,25^. The gating strategy used for the analyses including that for the negative controls is shown in Supplemental Fig. 1.

### Mixed lymphocyte reaction suppression assay

Freshly isolated responder PBMC autologous to the Tregs were stimulated with freshly isolated “donor”-specific irradiated PBMC from the B cell donor or an irrelevant third party at a ratio of 1:1 in U-bottom 96-well plates in triplicate. Tregs or irradiated R-PBMC (control) were added at indicated modulator: responder ratios at the initiation of the suppression assays. Even though the “none” control is used widely, the true control should be where the same number of modulator control cells are added to the culture but without observing any inhibitory effect. If conventional T cells from the same donor were to be expanded in the same way as Tregs, they would generate cytotoxic effector T cells that would then cause the lysis of the stimulator cells of the inhibition experiment^53^ resulting in either non-responsiveness or partial response. Therefore, it was found that the best control would be equivalent number of either irradiated or non-irradiated responder PBMC added as the modulator control^54^. The cells were irradiated at 3,000 rads. After 7 days, ^3^H-thymidine was added to the cultures during the final 16-20 hours and incorporation of ^3^H-thymidine was used to measure proliferation. The back-response CPM given by the Tregs or control modulators at the tested doses were also assessed in separate cultures (Rx + Dx/Ix + AgTregs/Rx) and were subtracted from (R + Dx/Ix + AgTregs/Rx) experimental cultures to obtain the delta CPM (Δ CPM). The mixed lymphocyte reaction (MLR) inhibition was quantified as:

$$ \% \,{\rm{inhibition}}=1-(\frac{{\rm{\Delta }}\,{\rm{CPM}}\,{\rm{in}}\,{\rm{presence}}\,{\rm{of}}\,{\rm{Tregs}}}{{\rm{\Delta }}\,{\rm{CPM}}\,{\rm{in}}\,{\rm{presence}}\,{\rm{of}}\,{\rm{Rx}}\,{\rm{controls}}})\times 100$$

### Treg-MLR (Infectious tolerance assay)

The ability of expanded Tregs to generate new Tregs infectiously from naïve responder cells was measured using the “Treg-MLR” as described previously^24^. Briefly, MLR cultures were set up with “recipient” CFSE-labelled responders stimulated with irradiated (3,000 rads) and PKH26-labelled donor-specific or allo-irrelevant PBMC. PKH26-labelled modulator cells composed of either Tregs or R-PBMC treated in an equivalent manner to serve as controls were then added at modulator: responder ratios of 1:10, 1:50, and 1:250. On day 7, flow cytometry was performed on the cultured cells after labelling with CD127-PE, CD4-ECD, CD25-PC7 (all from Beckman-Coulter), and FOXP3-PC5 (eBioscience). PKH26-labelled modulators and any surviving stimulators as well as CD127-PE^+^ responder cells were gated out, and CD4^+^ cells that proliferated (as determined by reduced CFSE expression) were analyzed for CD25 and FOXP3 expressions. Thus, the percentage of CD4^+^CD127^−^CD25^High^FOXP3^+^ cells that were newly generated in the proliferating responder cells was determined.

### Methylation Analysis

Cell pellets from whole Treg cultures without purification or depletion of residual stimulator B cells were sent to EpigenDx (Hopkinton, MA) for DNA extraction, bisulfite conversion, and pyrosequencing. Percent methylation was calculated as (% methylated cytosine)/(% methylated cytosine + unmethylated cytosine). All Treg cultures were from female donors; however, no correction for X-linked inactivation^29^ was made when the results were plotted.

### TCR repertoire analysis

Cell pellets of 0.5 × 10^6^ from 21-day expanded Tregs cultures (from 3 different AgTregs and 3 different polyclonal expansions) and the Day 0 CD4^+^CD25^+^ starting Treg culture (in duplicate) were sent to ArcherDx (Boulder, CO) for survey level TCRβγ Immunoverse Analysis. Normal PBMC was used as a control to ensure that the largest number of T cell clones were identified in the assay. The RNA were extracted with Qiagen RNeasy and quantified via Qubit. Libraries were prepared with sample input normalized by volume (20uL eluate) and the TCRβγ Immunoverse analyses were performed. The data including the number of clones, number of clonotypes and the diversity were analyzed using algorithms developed by ArcherDx.

### Statistical Analysis

Paired Student T-tests and Wilcoxon signed rank tests for parametric and nonparametric calculations respectively were used. P values of ≤0.05 were considered statistically significant and indicated by * in the Figures. If not otherwise mentioned, the data are shown as mean ± SD.

### Data Availability

The datasets generated during and/or analyzed during the current study are available from the corresponding author on reasonable request.

## Electronic supplementary material

Supplementary Information
