# Supplementary material for: Generation and Characterization of Alloantigen-Specific Regulatory T Cells For Clinical Transplant Tolerance
Source: Sci Rep. 2018 Jan 18;8:1136. doi: 10.1038/s41598-018-19621-6 (PMC5773708; doi:10.1038/s41598-018-19621-6)
Supplement: Supplementary file 1 — Supplementary Information [file 41598_2018_19621_MOESM1_ESM.pdf]

ENERATION AND CHARACTERIZATION OF ALLOANTIGEN-SPECIFIC REGULATORY T CELLS FOR CLINICAL TRANSPLANT TOLERANCE

James M. Mathew[1, 2,*], Jessica L. Heinrichs[1], Scott T. McEwen[1,4], Iwona Konieczna[1], Arjun Chakraborty[1], Xuemei Huang[1], Jie He[1], Lorenzo Gallon[1, 3], Richard S. Kornbluth[5] & Joseph R. Leventhal[1]

[1]Department of Surgery - Comprehensive Transplant Center, [2]Department of Microbiology-Immunology, [3]Department of Medicine- Nephrology; Northwestern University Feinberg School of Medicine; [4]Ann & Robert H. Lurie Children's Hospital; Chicago; IL.

[5]Multimeric Biotherapeutics, Inc.; La Jolla, CA

Corresponding author: James M. Mathew, PhD; Email: james-mathew@northwestern.edu

Supplementary Information

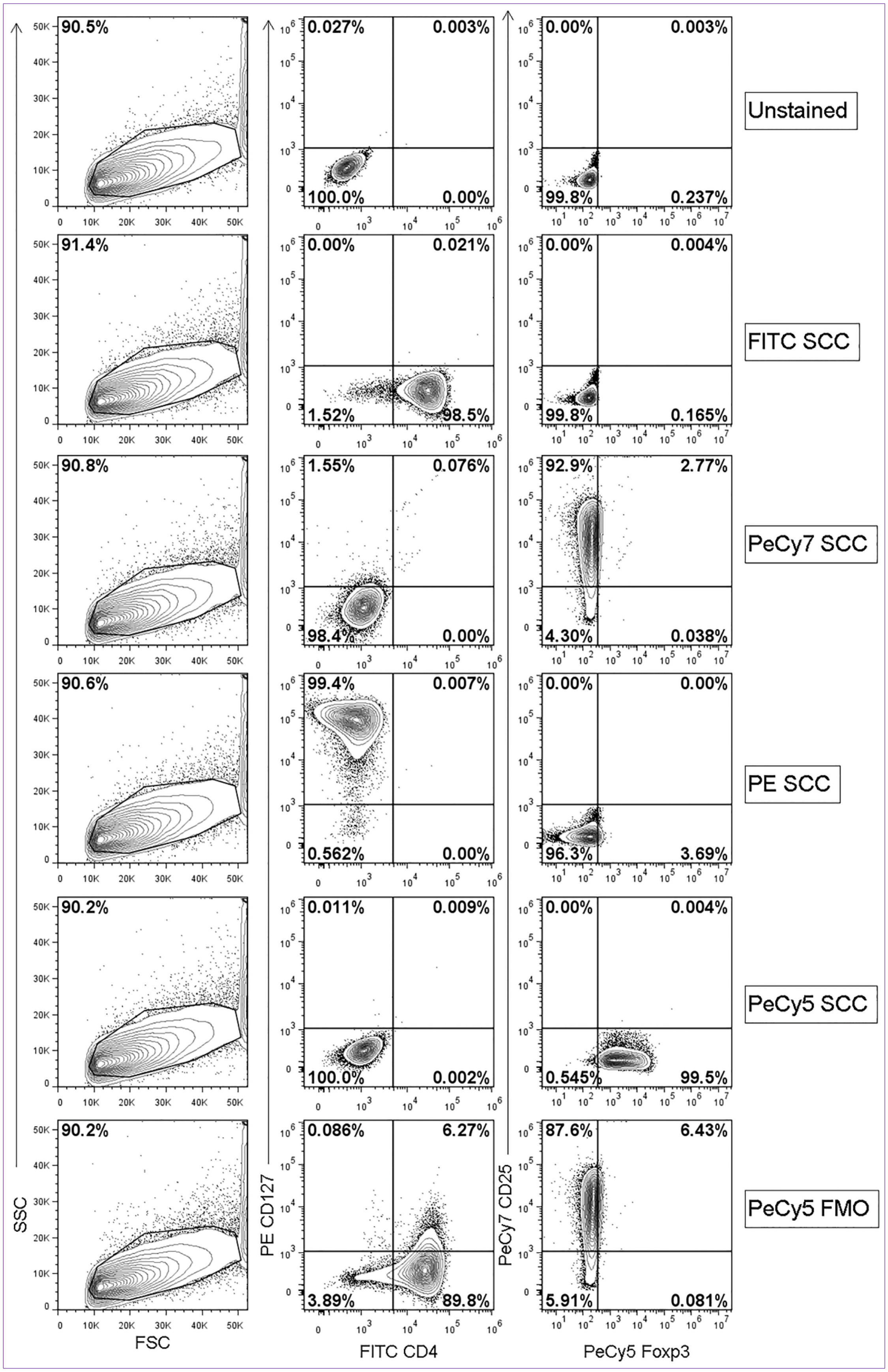
Unstained
FITC SCC
PeCy7 SCC
PE SCC
PeCy5 SCC
PeCy5 FMO
SSC
FSC
PE CD127
FITC CD4
PeCy7 CD25
PeCy5 Foxp3
90.5%
0.027%
0.003%
100.0%
0.00%
0.00%
0.003%
99.8%
0.237%
91.4%
0.00%
0.021%
1.52%
98.5%
0.00%
0.004%
99.8%
0.165%
90.8%
1.55%
0.076%
98.4%
0.00%
92.9%
2.77%
4.30%
0.038%
90.6%
99.4%
0.007%
0.562%
0.00%
0.00%
0.00%
96.3%
3.69%
90.2%
0.011%
0.009%
100.0%
0.002%
0.00%
0.004%
0.545%
99.5%
90.2%
0.086%
6.27%
3.89%
89.8%
87.6%
6.43%
5.91%
0.081%

Supplementary Figure 1: *Gating Strategy Used for Intracellular Staining of Expanded Tregs* (representative experiment)***:*** To accurately set positive and negative gates, unstained control, single color controls (SCC), and Fluorescence minus one (FMO) were used in all experiments.

Initially, the gates were set using unstained expanded Tregs (Top). They were then further adjusted using single color controls of CD4-FITC, CD45-PE (as CD127 is not expressed on Tregs), FOXP3-PeCy5 or CD25-PeCy7 (rows 2-5). Since intracellular staining for FOXP3 results in a spread population, the final positive and negative gate was confirmed by comparing the FOXP3-PeCy5 SCC and the Pecy5 FMO (FMO = cells stained with CD4-FITC, CD127-PE and CD25- PeCy7 but without the FOXP3-Pecy5; bottom). The protocol set-up was further verified by using the full complement of fluorochromes in one tube (not shown). At least 10,000 events were acquired for all control, SCC and FMO tubes.

Our use of the actual expanded Tregs allowed a better understanding of the natural expression of each molecule that could not have been obtained if we had used artificial beads.

Similar unstained control, SCC and verification strategy was used for the flow cytometric analyses of B cells shown in Figure 1.
